# Supplementary material for: Intra- and intergenerational costs of handicapping in the Saffron Finch (Sicalis flaveola), a thraupid with delayed plumage maturation
Source: PLoS One. 2025 Sep 12;20(9):e0331227. doi: 10.1371/journal.pone.0331227 (PMC12431269; doi:10.1371/journal.pone.0331227)
Supplement: S1 Table — (DOCX) [file pone.0331227.s001.docx]

**S1 Table**. Lack of correlations in physical condition between mates, in experimentally handicapped pairs and control pairs of Saffron Finches.

| **Comparison** | **Pearson´s correlation coefficient** | ***P*-value** |
| --- | --- | --- |
| **Handicapped ASY males vs. their female mates** | -0.22 | 0.435 |
| **Handicapped females vs. their mate ASY males** | 0.11 | 0.720 |
| **Non-handicapped ASY males vs. their mate females** | -0.13 | 0.596 |
| **Handicapped SY males vs. their female mates** | 0.14 | 0.668 |
| **Handicapped females vs. their non-handicapped SY mates** | 0.01 | 0.985 |
| **Non-handicapped females vs. their non-handicapped SY males** | 0.12 | 0.641 |
