# Supplementary material for: Intra- and intergenerational costs of handicapping in the Saffron Finch (Sicalis flaveola), a thraupid with delayed plumage maturation
Source: PLoS One. 2025 Sep 12;20(9):e0331227. doi: 10.1371/journal.pone.0331227 (PMC12431269; doi:10.1371/journal.pone.0331227)
Supplement: S2 Table — (DOCX) [file pone.0331227.s002.docx]

**S2 Table**. Average weight difference (weight at day 10 minus weight at day 1 of nestlings’ life, mean ± S.E and 95% CI) of adults of both sexes attending experimental and control nests. Numbers in bold font indicate significant p-values (< 0.05).

| **Comparison** | **Average weight difference** | | |
| --- | --- | --- | --- |
|  | **Mean ± *S.E (g).*** | **95% CI** | ***p-value*** |
| **Handicapped ASY males** | - 0.63 ± 0.31 | - 1.24 : - 0.02 | 0.153 |
| **Non Handicapped ASY males** | - 0.53 ± 0.22 | - 0.96 : - 0.10 |  |
| **Handicapped females mated with ASY males** | - 0.75 ± 0.19 | - 1.12 : - 0.38 | 0.263 |
| **Non Handicapped females mated with ASY males** | - 0.96 ± 0.09 | - 1.13 : - 0.79 |  |
| **Handicapped SY males** | - 0.30 ± 0.17 | - 0.63 : - 0.03 | 0.471 |
| **Non Handicapped SY males** | - 0.41 ± 0.26 | - 0.92 : -0.10 |  |
| **Handicapped females mated with SY males** | - 1.75 ± 1.16 | - 3.99 : - 0.49 | **0.011** |
| **Non Handicapped females mated with SY males** | - 0.97 ± 0.11 | - 1.19 : - 0.75 |  |
