# Supplementary material for: Intra- and intergenerational costs of handicapping in the Saffron Finch (Sicalis flaveola), a thraupid with delayed plumage maturation
Source: PLoS One. 2025 Sep 12;20(9):e0331227. doi: 10.1371/journal.pone.0331227 (PMC12431269; doi:10.1371/journal.pone.0331227)
Supplement: S3 Table — (DOCX) [file pone.0331227.s003.docx]

**S3 Table**. Results of the Generalized Linear Mixed Model comparing the nest temperature in experimental and control nests, for all treatments.

| **Comparison** | **Nest temperature (°C)** | | | |
| --- | --- | --- | --- | --- |
|  | **Wald’s statistic** | ***F*** | ***P*-value** | **Mean ± *S.E.*** |
| **Handicapped ASY males vs. control** | 0.88 | 0.78 | 0.82 | 32.4 ± 1.3 |
| **Handicapped females mated with ASY males vs. control** | 0.14 | 0.47 | 0.39 | 31.9 ± 1.7 |
| **Handicapped SY males vs. control** | 0.63 | 0.15 | 0.62 | 33.4 ± 1.1 |
| **Handicapped females mated with SY males vs. control** | 0.55 | 0.54 | 0.16 | 31.1 ± 2.1 |
